# The assessment of the sexuality of patients with a borderline personality disorders based on their 2D:4D digit ratio

**DOI:** 10.1093/sexmed/qfaf006

**Published:** 2025-02-15

**Authors:** Justyna Holka-Pokorska, Adam Kucharski

**Affiliations:** Department of Pharmacology and Physiology of CNS, Institute of Psychiatry and Neurology, 02-957 Warsaw, Poland; Faculty of Psychology, University of Szczecin, 70-453 Szczecin, Poland

**Keywords:** borderline personality disorder, female sexuality, sexual response, 2D:4D digit ratio, female sexual arousal

## Abstract

**Background:**

The hormonal composition of amniotic fluid during prenatal development, particularly the androgen-to-estrogen ratio, may influence neuronal differentiation related to sexual response patterns and the capacity to control impulsive sexual behaviors in later life.

**Aim:**

This study aims to assess sexual behaviors and characterize sexual responses in women with borderline personality disorder (BPD) compared to a control group.

**Methods:**

The study included 33 women diagnosed with BPD and 56 women in a control group. BPD diagnoses were based on clinical psychiatric evaluation and the Structured Clinical Interview for DSM-IV. Elements of sexual response were measured using the Arizona Sexual Experience Scale (ASEX). The digit ratio (2D:4D) served as a biomarker for testosterone and estrogen exposure during early prenatal development.

**Main outcomes:**

ASEX results were analyzed in the categories of “desire,” “arousal,” and “vaginal lubrication” subscales, along with the 2D:4D digit ratio for both hands.

**Results:**

Lower values for the right–hand digit ratio were observed in the BPD group compared to the control group (0.989, SD = 0.034 vs. 1.016, SD = 0.039; *P* = 0.0014), potentially indicating higher prenatal testosterone levels. Significant correlations were found in the BPD group between the right–hand digit ratio and scores on the ASEX subscales, specifically “sexual arousal” (*r* = 0.406, *P* = 0.019) and “vaginal lubrication” (*r* = 0.362, *P* = 0.038).

**Clinical implications:**

These results may support biological hypotheses regarding the origins of sexual dysfunction in women with BPD.

**Strengths and limitations:**

This study is a pioneering attempt to explore the indirect impact of early amniotic hormone composition on the neurobiological conditioning of sexual response and behavior in women with BPD. Limitations include the preliminary nature of the findings, a small sample size, and results that may not be generalizable across all genders.

**Conclusions:**

Physiological aspects of sexual response, such as arousal and vaginal lubrication, in women with BPD appear to be significantly correlated with prenatal testosterone levels, as indicated by the 2D:4D digit ratio.

## Introduction

Borderline personality disorder (BPD) stands out among psychiatric disorders due to its complex symptomatology, characterized by instability in affect, relationships, and self–image. It affects approximately 1%-2% of the general population, with notably higher prevalence rates among clinical populations, such as psychiatric inpatients or outpatients.^1^ Research concerning the biomarkers of pathological personality traits and the physiology of the sexual response in women with BPD is still limited.

Previous research and clinical practice show that patients with BPD display a range of sexual problems. BPD women experience higher than normal sexual dissatisfaction, depression and orgasm dysfunctions but at the same time higher than normal sexual assertiveness, higher self–esteem, greater erotophilic attitude, and eagerness to engage in sexual contacts with partners from outside their relationships.^2^ Additionally, among this group, risky sexual behaviors and impulsive sexual reactions could be considered a form of a strategy to continue to keep their partner.^3^ The impulsive sexual activity is reflected in the uncontrolled initiation of new sexual relationships and a high number of sexual encounters. Comparative studies have shown that persons with BPD exhibit a higher lifetime number of sexual partners compared to non–BPD persons.^4^ In patients with BPD, there is also observed the opposite pattern of avoidance or withdrawing from the sexual activity.^5^

Research concerning the biomarkers of pathological personality traits and the physiology of the sexual response in women with BPD is still limited. One of the biological markers of sexually dimorphic traits and human sexuality is testosterone, mostly known for its role in the development of sex organs and physical maturation during puberty. Testosterone and other steroid hormones also operate in the prenatal period, which is a critical time for sex steroids to shape the brain circuits and normal central nervous system functions later in life.^6–8^ In humans, a critical period for the organization of the brain is thought to be between weeks 8 and 24 of gestation.^9^ A method of examination of hormones in amniotic fluid and then relating these hormones to behavior later in life, was first proposed by Finegan et al.^10^ Through direct measurements it was determined that testosterone levels are peaking high in the fetal serum between weeks 12 and 18 of pregnancy. There has been considerable interest in studying indirect indicators of prenatal testosterone exposure in the uterine environment in relation to behaviors presented postnatally by children and adults. One of the proposed indicators is the length ratio of the second to fourth finger (2D:4D digit ratio) and it was linked to a range of sexually dimorphic traits and behaviors, including aggression, mating preferences, and sexual orientation.^11^ According to Manning et al., because of the ethical constraints in the measurement of the effect of fetal testosterone and fetal estradiol on 2D:4D directly, indirect correlations of the balance between sex steroids and the 2D:4D could be valuable in investigating organizing effects on traits connected with sexual behavior.^12^ In most studies, the digit ratio shows positive correlations with typical female behaviors and negative correlations with typical male behaviors.^13^ The 2D:4D digit ratio was associated with variations in sexual response patterns and behaviors among women, with lower ratios (indicating higher prenatal testosterone exposure) being correlated with increased sexual desire, arousal, and satisfaction.^12^ There are other studies of individuals with disorders of sexual differentiation like congenital adrenal hyperplasia, Klinefelter syndrome and Turner syndrome finding the correlations of 2D:4D digit ratio with early gonadal steroid exposure^14–17^, which also revealed the effects of fetal masculinization or feminization.

Given the potential role of prenatal hormone exposure in shaping sexual behaviors and response patterns, investigating the association between digit ratio and sexual functioning in women with BPD holds promise for elucidating the underlying biological factors contributing to their sexual difficulties. Understanding the relationship between digit ratio and sexual response in this population may have implications for tailoring therapeutic interventions. Little is known about the character and variations of the sexual response itself in women with BPD and its relationship between digit ratio and sexual functioning, though sexual impulsivity is one of the categories that define the clinical diagnosis of BPD.

The digit ratio assessment of early gonadal steroid exposure has unique strengths as an indirect method of studying the fetal environment that might influence the shaping of neuronal circuits, but also has notable weaknesses as the method of not fully understood reliability and reproducibility.^14^ Therefore, the results of studies using 2D:4D ratio as a metric of sex steroids levels during the prenatal period should be treated with caution until converging evidence is available from multiple methods and authors.

Under the assumption that the 2D:4D digit ratio is a marker of levels of testosterone and estradiol in prenatal development, the current study explores the sexual functioning of adult women with BPD. By examining dependencies between digit ratio and sexual desire, arousal, orgasm, and sexual satisfaction, we aim to shed light on the underlying mechanisms contributing to sexual problems observed in this population. The results obtained with the digit ratio in the BPD group were compared with the control sample.

## Aim

This study describes biologically conditioned sexual behavior specific to borderline women as contrasted with women without the BPD diagnosis. It was assumed that the hormonal composition of amniotic fluid in prenatal development, especially the androgen-to-estrogen ratio modulates the differentiation and development of neurons related to the patterns of the sexual response and the ability to control sexual impulsive behaviors later in life. The androgen-to-estrogen ratio in prenatal development was indirectly measured by its correlation to the 2D:4D ratio.

The following study questions were posed:

1. Could the androgen-to-estrogen ratio in early prenatal development condition sexual activity and sexual response in women with BPD?
2. Do women with BPD exhibit the same sexual behaviors as women without BPD?

## Methods

### Study setting and study sample

This study was conducted between 2014 and 2019 with a group of women diagnosed with BPD and with women from a control group without BPD. The BPD group were recruited from the group of patients receiving any psychiatric treatment for the BPDs. The healthy controls were recruited from adult women who have not previously received psychiatric treatment based on the public announcement about the study.

Inclusion criteria for the BPD group included the following: age over 18 y/o, the ability to provide informed consent to participate in the study, a history of psychiatric treatment for BPD, diagnosis of BPD based on the diagnostic criteria of ICD-10 and the SCID-II for DSM-IV.^18^

Inclusion criteria for the control group included the following: age over 18 y/o, the ability to provide informed consent to participate in the study, no history of psychiatric treatment for personality disorders, and no diagnosis of BPD based on the diagnostic criteria of ICD-10 and the SCID-II for DSM-IV.^18^

Data from 35 women with BPD and 56 women without BPD were used for the analysis of sexual functioning based on self–assessment questionnaires. Two women provided only partial data and therefore were not included in all analyses. In the statistical analysis of sexual functioning based on the Arizona Sexual Experience (ASEX) data from 33 BPD and 56 non–BPD women were used. This research was approved by the Bioethics Committee at the Institute of Psychiatry and Neurology in Warsaw. The study adheres to the tenets of the *Declaration of Helsinki*.

BPD was diagnosed based on a psychiatric interview, a clinical psychiatric examination, and a clinical interview SCID-II for DSM-IV.^18^ SCID-II is a partially structured clinical interview that allows for an assessment of 12 personality disorders classified by AXIS II of DSM-IV.

### Demographic factors

The participants of the study were asked to provide information on their age, marital status, education, place of living, sexual orientation, and lifetime number of sexual partners.

### 2D:4D digit ratio measure

According to Manning, the length of the 4th digit indicates prenatal exposure to androgens, while the length of the 2nd digit is indicative of estrogen exposure in prenatal development.^19^ The digit ratio is formed during prenatal development, about the 14th week, and does not change in later ontogenetic development. The 2D:4D digit ratio is a dimorphic marker and equals 0.98 for men, and 1.00 for women.^19–21^ A lower digit ratio corresponds to high prenatal testosterone levels and low prenatal estrogen levels, whereas a higher digit ratio is associated with low prenatal testosterone levels and high prenatal estrogen levels.^22^

The digit ratio was used as a biomarker for prenatal testosterone and prenatal estrogen levels during early prenatal development. The digit ratio was measured by both authors of the study on the ventral, palm–side of both hands, from the midpoint of the bottom crease to the tip of the second (index) finger and fourth (ring) finger, using an electronic calliper. For each finger, two measures were taken, and their mean was calculated. Finally, the D2:D4 ratio was calculated, by dividing the length of the index finger of a given hand by the length of the ring finger of the same hand.

### Measures of sexual functioning based on ASEX Scale

ASEX Scale^23^ was used to assess the sexual response of participants.

**ASEX** is a questionnaire available in two versions, for men and for women. It consists of five questions that aim at quantitative evaluation for the following aspects: sex drive, arousal, vaginal wetness/penis erection, ability to reach orgasm, and satisfaction. Each respondent is asked to rate sexual functions by assigning them a score from 1 to 6 points, the score of “1” corresponding to “Extremely strong/easily”, the score of “2” corresponding to “very strong/easily”, the score of “3” corresponding to “somewhat strong/easily”, the score of “4” corresponding to “somewhat difficult/weak”, the score of “5”corresponding to “very difficult/weak”, and the score of “6” corresponding to “absent/never”. The total score of the sum of five questions ranges between 5 and 30 points, with the higher score indicating a more severe sexual dysfunction and the lower score indicating more physiological, flexible sexual response.^23^

### Statistics

Data were analysed using SPSS Statistics for Windows, Version 23.0 (IBM SPSS Statistics for Windows, Version 23.0. Armonk, NY: IBM Corp). Descriptive data were compared using the Mann–Whitney U test to study differences between two groups. A relationship between quantitative variables was calculated using Spearman’s correlation coefficient. The statistical significance threshold was set to *P* = 0.05.

## Results

### Demographic characteristics

Our study cohort comprised 33 women diagnosed with BPD and 56 women without BPD, with 89 women in total. Hypothesis 0 in the analysis assumed that the distributions under test are the same. Detailed results concerning demographics are presented in Table 1.

**Table 1:** Main demographic factors of women with BPD and from the control group without the BPD diagnosis.

	Results of group comparison	Patients with BPD	Control Group: without BPD
*N* = 89		*N* = 33	*N* = 56
Mean age (y/o)	*P* = 0.0001^**^	25.6 +/− 1.1	20.6 +/− 0.3
Marital status Married Widow Separated Single No answer	*P* = 0.161	0 0 2 (6.1 %) 31 (93.9 %) 0	0 0 0 56 (100%) 0
Education: Secondary school Vocational school High School Vocational High School Bachelor’s degree University Degree	*P* = 0.042^*^	4 (12.1 %) 1 (3.0 %) 15 (45.5 %) 0 5 (15.2 %) 8 (24.2 %)	0 0 52 (92.8 %) 2 (3.6 %) 1 (1.8 %) 1 (1.8 %)
Place of living: Rural area City less than 50.000 City 50.000-200.000 Regional city (200.000-500.000) City over 500.000	*P* = 0.001^**^	2 (6.1 %) 4 (12.1 %) 0 14 (42.4 %) 13 (39.4 %)	7 (12.5 %) 13 (23.2 %) 7 (12.5 %) 27 (48.2 %) 2 (3.6 %)
Sexual orientation: Heterosexual Homosexual Bisexual Asexual No answer	*P* = 0.258	20 (60.6 %) 1 (3 %) 10 (30.3 %) 1 (3 %) 1 (3 %)	41 (73.2 %) 2 (3.6 %) 10 (17.8 %) 2 (3.6 %) 1 (1.8 %)

Abbreviation: BPD, borderline personality disorder

The mean age of the BPD group was 25.6 ± 1.1 and 20.6 ± 0.3 years in the control group. A statistically significant difference in mean age was observed between the sub–groups based on a two–sample *t*-test, assuming unequal variances with probability *P*(*T* ≤ *t*) = 0.0001.

Most participants in the study group were single, with only two women in the BPD group reported as separated. The groups differed significantly in terms of their educational degree (*P* = 0.042). The most common educational attainment reported by participants in the entire group was “high school” (BPD 92.8% vs non–BPD 45.5%). Subsequently, a university degree was reported by 24.2% of the BPD group compared to 1.8% in the control group.

Both groups differed in terms of their place of living (*P* = 0.001). Participants in the BPD group predominantly resided in larger cities (39,4% vs 3.6%) with populations exceeding 500.000, and (42.4% vs 48.2%) in cities with populations 200.000-500.000. Respondents in the control group, (12.5% vs 0%) resided in cities with populations ranging from 50.000 to 200.000, (23.2% vs 12.1%) in small cities with populations below 50.000, and (12.5% vs 6.1%) in rural areas.

Most women in the BPD and non–BPD groups identified themselves as heterosexual (60.6% vs 73.2%), while (30.3% vs 17.8%) identified themselves as bisexual, (3.0% vs 3.6%) as homosexual, and only (3.0%vs 3.6%) as asexual.

In summary, it can be noticed that both groups differed significantly in terms of demographic characteristics like mean age, level of education, and place of residence. The mean age of the participants from the BPD group was higher than those from the non–BPD group was 25.6 ± 1.1 and 20.6 ± 0.3 years in the control group. The groups did not differ in terms of declared sexual orientation. Among the entire study group, most people declared heterosexual orientation, although over 30% of the BPD group and almost 18% of the non–BPD group declared bisexual orientation.

**Figure 1:**
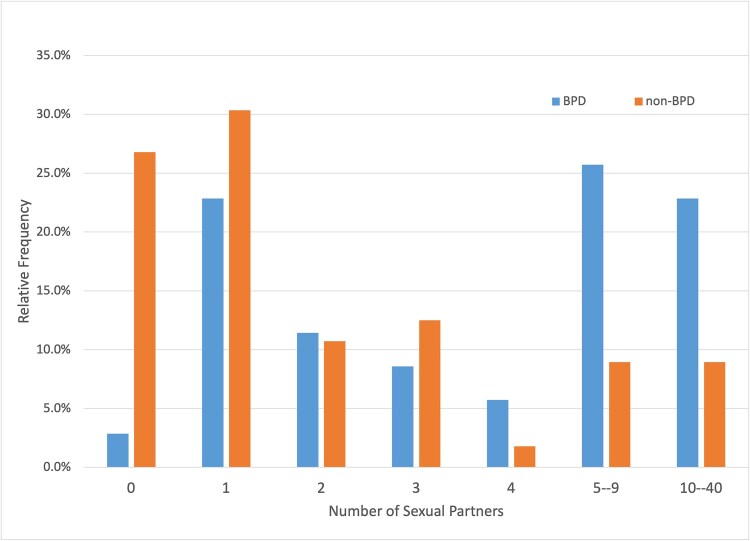
Lifetime number of sexual partners in the BPD group and non–BPD group. Abbreviation: BPD, borderline personality disorder.

To better understand the sexual functioning patterns of women in the study groups, an estimate of lifetime number of sexual partners was requested. It was found that the study group exhibited variability in terms of number of sexual partners. Some participants chose not to disclose their relationship history. Women with BPD declared more sexual partners (Avg = 6, SD = 6.2) then control group (Avg. = 3, SD = 4.8) (U = 471,50; *P* < 0.001) (see Figure 1).

### Analysis of 2D:4D finger ratio

Our first step was a comparative analysis of the 2D:4D ratio (index finger to ring finger) in woman with BPD as compared to woman without BPD. As the variables in question were not normally distributed, we conducted the Mann–Whitney U test. A statistically significant difference was found between the groups for the relevant digit ratio on the right hand (U = 546.5; *P* < 0.0014).

However, the differences in the 2D:4D ratio of the left hand between the BPD group and the control group turned out to be statistically insignificant (U = 773; *P* < 0.2).

No additional significant differences were observed between hands or groups (see Table 2). Based on these results, lower values of the digit ratio were observed for borderline women, which could point to higher levels of prenatal testosterone for women with BPD.

**Table 2:** Comparison of the mean of ranks of the 2D:4D finger ratio in the right and left hands in women with BDP disorders and in women without BDP disorders.

	*N* = 89	Hand choice	Mean of ranks group comparison	Results of group comparison for a hand	Mean of ranks hand comparison	Results of hand comparison in a group
Women without BPD	56	Right	51.7	U = 546.5; *P* < 0.0014^**^	59.0	U = 1426; *P* < 0.41
		Left	47.7	U = 773; *P* < 0.2	54.0	U = 1426; *P* < 0.41
Women with BPD	33	Right	33.6	U = 546.5; *P* < 0.0014^**^	36.3	U = 451; *P* < 0.23
		Left	40.4	U = 773; *P* < 0.2	30.67	U = 451; *P* < 0.23

Abbreviation: BPD, borderline personality disorder

The results of 2D:4D ratio for the left and right hand are presented in Table 3 with the following variables: Average, SD, Min and Max Values.

**Table 3:** Values of the digit ratio for the right and left hands.

	BPD group		Non–BPD group	
2D:4D finger ratio	Righthand	Left hand	Right hand	Left hand
Average	0.989	0.996	1.016	1.006
SD	0.034	0.034	0.039	0.038
MIN values	0.915	0.913	0.961	0.900
MAX values	1.057	1.059	1.135	1.074

We also compared the mean values of the 2D:4D ratio of the right hand of BPD and non–BPD participants. The analysis of the distributions using the Welch *t*-test revealed significant differences between both groups (*P* < 0.001). The means of 2D:4D finger ratio of the right hand turned out to be statistically significant based on the Mann–Whitney U test (U = 546.5; *P* = 0.0014^**^). The distribution of the mean values of the 2D:4D ratio of the left hand did not differ statistically significantly based on the Welch *t*-test (*P* < 0.235). The differences in 2D:4D finger ratio of the left hand between the BPD and non–BPD group did not differ significantly based on Mann–Whitney U test (U = 773; *P* = 0.2).

**Figure 2:**
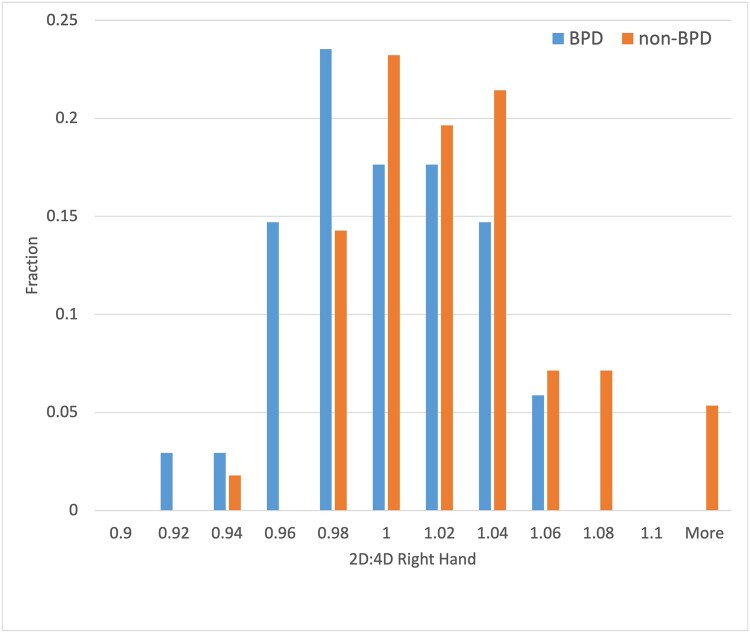
Distributions of finger ratio 2D:4D of the right hand in BPD and non–BPD groups normalized to the equal number of participants. Abbreviation: BPD, borderline personality disorder.

Histogram in Figure 2 illustrates a comparison of results of the finger ratio for BPD and non–BPD groups on the right hand of participants. The distributions of the 2D:4D finger ratio were different with the following results of the Mann–Whitney U test performed on them (U = 546.5, *P* = 0.0014). The statistically significant result of this comparison confirmed that the two distributions under test are different. The values of the 2D:4D finger ratio of the BPD group are shifted towards lower values than for the non–BPD group.

### ASEX results

**Figure 3:**
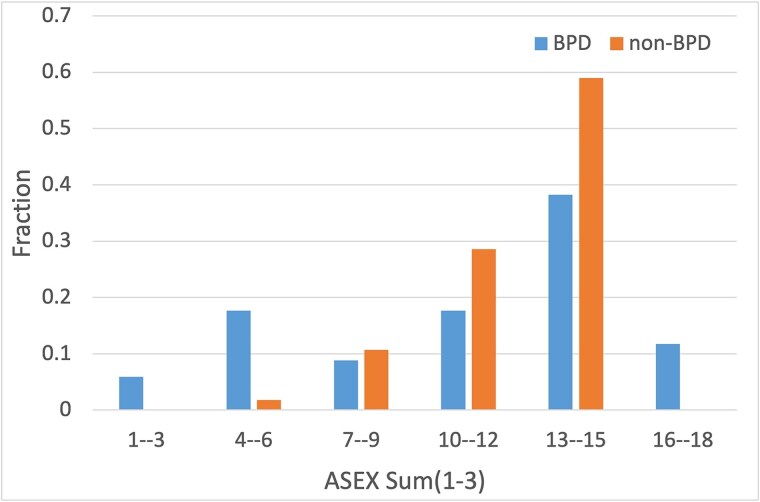
Normalized distributions of sums of scores to questions 1, 2, and 3 for BPD and non–BPD groups.

The ASEX study was performed to better understand the sexual functioning among BPD and non–BPD participants. In the analysis of the ASEX questionnaire we considered only answers to questions 1, 2, and 3. ASEX questions 4 and 5 relate to the most recent sexual activities of participants and since not all respondents were sexually active during the last week before filling out the questionnaire, answers to these questions were not analysed. The results of the answers to questions 1, 2, and 3 and their sum are analyzed and the sum of scores for three questions is presented in the histogram in Figure 3. More BPD than non–BPD respondents provided an answer with the value of “1” (“Extremely strong sex drive”) to question 1 “How strong is your sex drive?”. For other scores, and especially with values 4, 5, and 6, there were more answers for non–BPD than BPD respondents.

Among the answers to the question 2 “How easily are you sexually aroused?” the participants from the BPD group are found in all categories of answers, but they dominate the non–BPD group in categories “1” and “2” (“Extremely easy” or “very easy sexual arousal”) and “5” and “6” (“very difficult sexual arousal” and “never aroused”). However, non–BPD group members dominate the BPD group mainly in answers “3” and “4” (“Somewhat easy” and “Somewhat difficult”).

Among possible answers to the question 3, “How easily does your vagina become moist?”, at the lowest scores of “1” and “2” (“Extremely easy” or “Very easy vaginal moisture”), we can also find more entries for BPD than non–BPD participants.

Figure 3 presents the sum of scores to questions 1, 2, and 3, with scores below the value of 8 are dominated by the BPD over non–BPD participants.

#### Correlations of 2D:4D and ASEX results

**Figure 4:**
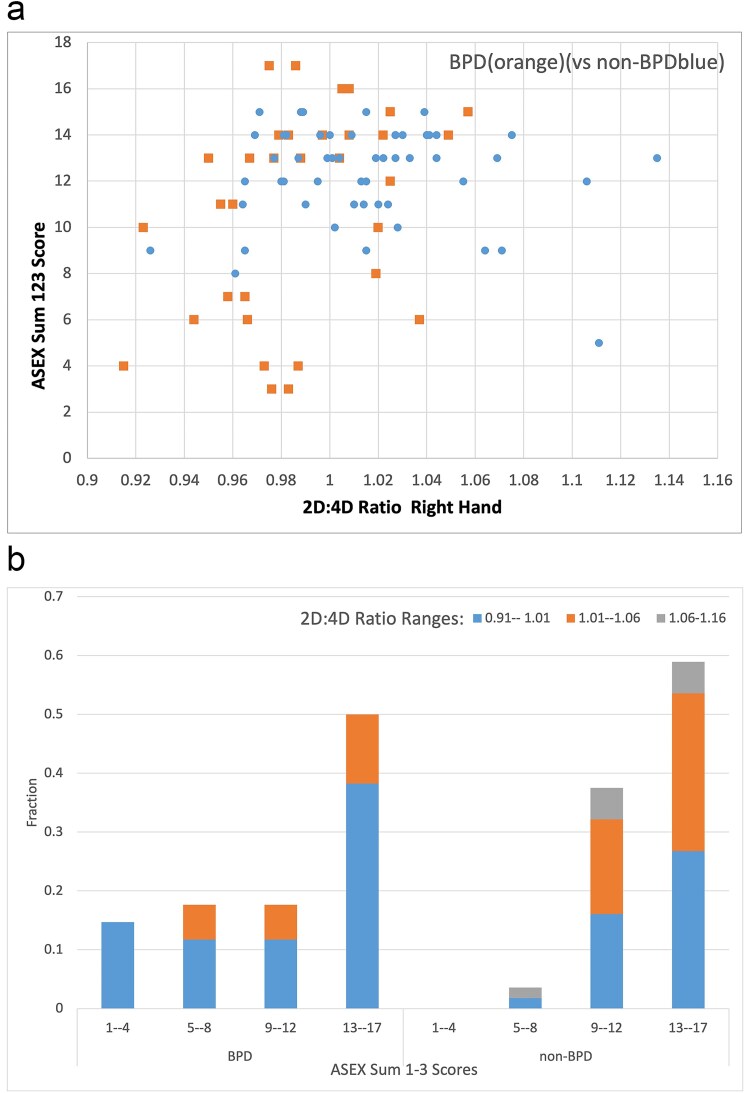
a. Correlations of the sum of answers’ scores to questions 1, 2, and 3 of ASEX and the 2D:4D digit ratio of the right hand in the BPD and non–BPD group. The sum of ASEX 1, 2, and 3 scores (y–axis) and the digit ratio (x–axis) are shown on the scatter plot on the left–hand side. In the histogram, on the right–hand side, the bins represent the normalized frequency of the sum of ASEX 1, 2, and 3 scores grouped: 1-2, 3-4, and 5-6 for three ranges (marked in blue, orange, and grey) of digit ratio for the BPD and non–BPD groups separately. b. Correlations of the sum of answers’ scores to questions 1, 2, and 3 of ASEX and the 2D:4D digit ratio of the right hand in the BPD and non–BPD group. The sum of ASEX 1, 2, and 3 scores (y–axis) and the digit ratio (x–axis) are shown on the scatter plot on the left–hand side. In the histogram, on the right–hand side, the bins represent the normalized frequency of the sum of ASEX 1, 2, and 3 scores grouped: 1-2, 3-4, and 5-6 for three ranges (marked in blue, orange, and grey) of digit ratio for the BPD and non–BPD groups separately. Abbreviations: ASEX, Arizona Sexual Experience Scale; BPD, borderline personality disorder.

To study the relationship between the basic elements of sexual response and digit ratio, the ASEX scale was used as a simple and frequently used scale in clinical studies designed for the assessment of the sexual response. The correlation analysis between the digit ratio and the results obtained in the first three ASEX responses and their sum was summarized in Table 4 and visually shown in Figure 4 for the sum of scores. The correlation results for answers to questions 2, 3, and the sum show the lack of responses with values “1”, “2”, and “3” in the group of non–BPD (left bottom corner of Figure 4). However, score values “1”, “2”, and “3” are observed only for women from the BPD group with low digit ratio. Score values “1”, “2”, and “3” correspond to the questionnaire answers expressing the most reactive sexual responses: “extremely strong/easy”, “very strong/easy”, and “somewhat strong/easy”. As the variables were not normally distributed, the correlation calculations were done using Spearman’s rank correlation coefficient method. There were no statistically significant correlations between the digit ratio and answers in questions 1-3 of ASEX for the non–BPD control group. For the women with BPD, statistically significant moderate positive correlations were found between the digit ratio and subjective arousal (question 2) (*r* = 0.406, *P* = 0.019^*^), as well as the digit ratio and the intensity of the physical sexual response measured as vaginal lubrication (question 3), (*r* = 0.362, *P* = 0.038^*^). As expected, the sum of the answers to questions 1-3 was also positively correlated for the BPD group (see Table 4).

**Table 4:** The correlation between the digit ratio (2D:4D), and the scores of self–reported responses to ASEX questionnaire on sexual desire, arousal, and physiological indicators of sexual arousal (vaginal arousal).

The digit ratio 2D:4D correlation with ASEX results		Sex drive	Arousal	Vaginal lubrication	Sum of all subscales
Women without BPD *N* = 56	Correlation coefficient	0.098	0.146	−0.100	0.034
	Significance (two–tailed)	0.472	0.282	0.462	0.803
Women with BPD *N* = 33	Correlation coefficient	0.275	0.406^*^	0.362^*^	0.420^*^
	Significance (two–tailed)	0.122	0.019	0.038	0.015

Abbreviation: ASEX, Arizona Sexual Experience Scale

^**^ *P* < 0.01; ^*^P < 0.05

## Discussion

Lower values for the 2D:4D digit ratio of the right hand were observed for the group of women with BPD as compared to the control group (mean = 0.988, SD = 0.033 vs mean = 1.015, SD = 0.039) **(See** Error! Reference source not found. and 5). It confirms the observations from the literature that the right 2D:4D digit ratio was described to be more sensitive to fetal sex hormones than the left 2D:4D digit ratio.^15^ Lower values of the digit ratio might indirectly indicate higher levels of prenatal testosterone in serum during early prenatal development for borderline patients as compared to the control group.

Prenatal activation of brain cells due to sex hormones is assumed to condition the sex differences in adult life, including a significant impact on a wide array of sexual behaviors, aggression, cognitive functioning as well as sexual identity and sexual orientation. Any factors which inhibit the interactions between the hormones and the emerging brain systems during pregnancy might permanently influence later behaviors in life.^24^^,^^25^

A few studies on BPD, however, were concerned with testosterone levels and morning levels of cortisol in saliva.^26^^,^^27^ They established a link between BPD and disrupted functions along the hypothalamic–pituitary–adrenal axis (HPA). A meta–analysis of over 800 studies on the features of stress response in people with BPD, with ultimately 37 studies on the functioning of the HPA axis, revealed that patients with BPD were likely to exhibit higher levels of cortisol (singular and continuous measurements) as well as blunted cortisol outputs following psychosocial stressors.^28^ The research carried out by Rausch et al. uncovered increased testosterone levels in saliva in both men and women with BPD, accompanied by higher cortisol levels in awakening response in women with BPD as compared with non–borderline volunteers.^29^ Also, raised levels of testosterone for patients with BPD were recorded in their hair, as compared to non–borderline individuals from a control group, which might be symptomatic of chronic heightened levels of testosterone in women with BPD.^26^ The research on steroid content in hair references a cumulative assessment of the differences in the levels for a given substance across the period of 6 months before the test.^30^ Therefore, such tests on the levels of hair–present testosterone in women with BPD show a 6-month increase in the levels of testosterone in the serum (before the test) as compared with a control group of non–BPD people.

To our knowledge, this study constitutes a pioneering attempt to evaluate the physiology of sexual response in women with BPD using a biological marker 2D:4D digit ratio, which is indirect characteristic of the hormonal composition that shapes the neurobiological development of the nervous system, using the ASEX questionnaire. In our study, statistically significant moderate positive correlations were found between the digit ratio and subjective arousal (ASEX question 2) (*r* = 0.406, *P* = 0.019^*^), as well as the digit ratio and the intensity of the physical sexual response measured as vaginal lubrication (ASEX question 3) (*r* = 0.362, *P* = 0.038^*^).We could claim that both, the tendency to have multiple partners and the type of sexual response for women with BPD were correlated with raised levels of testosterone in early prenatal development (cf. lower digit ratio).

Features of sexual response in women who qualified for our research were assessed using simplified method of female sexual response evaluation when compared to modern circular models of female sexual response.^31^^,^^32^ However, despite the simplified assumption underlying this tool, the scale in question do differentiate between the basic components of sexual response, which are present in the most popular and modern female sexual functioning models, such as sexual desire or sexual arousal both apparent and objective.^31^^,^^32^ The Rosemary Basson sexual response model comprises the following aspects of female sexual arousal: subjectively experienced excitement along with genital and non–genital arousal.^33^ A series of empirical findings confirmed two separate categories for female sexual response: the subjectively experienced sense of excitement and objectively measurable genital arousal.

Based on our findings we can draw the following conclusions. In women with BPD, the objective genital indicators of sexual arousal (as reported under “arousal” and “vaginal lubrication” in ASEX) were correlated with prenatal levels of sex steroids as evidenced by the digit ratio. Moreover, women with BPD seem to be more sexually active, which is confirmed by the higher number of partners as compared to the control group, as well as more sexually reactive, which is demonstrated by the results of the effectiveness of their sexual response. Prenatal testosterone levels in women with BPD correlated more strongly with the basic physiological elements of genital response as “arousal” and “vaginal lubrication”.

The main limitation of this study refers to a small sample size (*N* = 33 for BPD vs *N* = 56 for the non–BPD group). On the other hand, the number of participants is like the previous studies concerning the 2D:4D ratio in the psychiatric populations. The diagnosis of the BPD was made based on the SCID-II for DSM-IV and not on more recent SCID-5-PD, which was not available at the time when this research was planned. The results of our study are not generalizable for all genders because we studied only female participants.

Another important limitation of the study was the age difference between groups, which was found to be statistically significant. To control for the age variable, additional analyses were conducted to determine whether age played a significant role in the characteristics of genital response (assessed by the ASEX scale). These analyses confirmed that the characteristics of sexual response and the number of partners in the subgroup of BPD patients under 30 and over 30 were comparable to the characteristics of the overall BPD group. Our study was not aimed at assessing the social determinants of sexuality, but rather at evaluating the influence of sex steroid levels in the amniotic fluid on sexual response patterns exhibited later in life. According to the literature, the 2D:4D finger ratio remains constant for an individual throughout ontogeny and is therefore independent of the participants’ age.

## Conclusions

The features of sexual response in women with BPD have not been studied in relation to the prenatal biological marker of testosterone levels in the amniotic fluid. The current study is a pioneering effort at determining the indirect influence of the hormonal composition of amniotic fluid in early development on neurobiological conditioning of sexual response and sexual behavior in women with BPD.

Our findings indicate that women diagnosed with BPD did not differ significantly from the control group in the main patterns of sexual activity or declared sexual orientation. However, differences were observed in the number of sexual partners (*P* = 0.019). The BPD group engaged in sexual activities with different partners twice as frequently as the non–BPD group. Lower values for the right–hand digit ratio were observed in the BPD group compared to the control group (0.989, SD = 0.034 vs 1.016, SD = 0.039; *P* = 0.0014), which may indirectly suggest higher levels of prenatal testosterone. Significant correlations were found in the BPD group between the right–hand digit ratio and scores on ASEX categories such as “sexual arousal” (*r* = 0.406, *P* = 0.019) and “vaginal lubrication” (*r* = 0.362, *P* = 0.038). Women with BPD seem to be more sexually active, as confirmed by their higher number of partners compared to the control group, and more sexually reactive, as demonstrated by the effectiveness of their sexual response. Physiological elements of sexual response, like arousal and vaginal lubrication in BPD participants, appeared to be indirectly correlated with prenatal testosterone levels, as indicated by the 2D:4D digit ratio.

## Data Availability

The data is available upon a reasonable request from the corresponding author.
